# Isolation, Identification, and Characterization of a New Highly Pathogenic Field Isolate of *Mycobacterium avium* spp. *avium*

**DOI:** 10.3389/fvets.2017.00243

**Published:** 2018-01-15

**Authors:** Liangquan Zhu, Yong Peng, Junxian Ye, Tuanjie Wang, Zengjie Bian, Yuming Qin, He Zhang, Jiabo Ding

**Affiliations:** ^1^China Institute of Veterinary Drug Control, Beijing, China

**Keywords:** avian tuberculosis, *Mycobacterium avium* spp. *avium*, isolation, identification, characterization

## Abstract

Avian tuberculosis is a chronic, contagious zoonotic disease affecting birds, mammals, and humans. The disease is most often caused by *Mycobacterium avium* spp. *avium* (*MAA*). Strain resources are important for research on avian tuberculosis and vaccine development. However, there has been little reported about the newly identified *MAA* strain in recent years in China. In this study, a new strain was isolated from a fowl with symptoms of avian tuberculosis by bacterial culture. The isolated strain was identified to be *MAA* by culture, staining, and biochemical and genetic analysis, except for different colony morphology. The isolated strain was Ziehl-Zeelsen staining positive, resistant to p-nitrobenzoic acid, and negative for niacin production, Tween-80 hydrolysis, heat stable catalase and nitrate production. The strain had the DnaJ gene, IS*1245*, and IS*901*, as well. Serum agglutination indicated that the *MAA* strain was of serotype 1. The *MAA* strain showed strong virulence *via* mortality in rabbits and chickens. The prepared tuberculin of the *MAA* strain had similar potency compared to the *MAA* reference strain and standard tuberculin *via* a tuberculin skin test. Our studies suggested that this *MAA* strain tends to be a novel subtype, which might enrich the strain resource of avian tuberculosis.

## Introduction

Avian tuberculosis is a serious, chronic infectious zoonotic disease in poultry, pet, or captive birds, animals, and humans that is caused by *Mycobacterium avium* spp. *avium* (*MAA*), a subspecies of the *M. avium* complex (MAC). Avian tuberculosis was classified as a List B disease by the World Organization for Animal Health and as a third-class animal disease in China. Clinical manifestations in birds include emaciation, depression, and diarrhea, and the prominent feature is the formation of caseous tubercular nodules and granulomas with calcification in the liver, spleen, intestine, and bone marrow (1–3). Humans exposed to infected birds may acquire a zoonotic infection, particularly in immunocompromised people such as those with HIV (3, 4). Once fowls in poultry farms are infected, avian tuberculosis can persist for a long time and is difficult to eradicate, due to the chronic carrier state and shedding of bacteria by infected fowls (5, 6). This disease can have a serious economic impact associated with mortality, morbidity, and a reduction of egg production (1, 6). The best way is to quarantine and slaughter the infected ones (6, 7), as well as antibiotic treatment with the infected individuals (8, 9).

The MAC is a group of closely related non-tuberculous mycobacterial species and subspecies which includes both veterinary and opportunistic human pathogens. In addition to birds, MAC may also infect different animal species, such as swine, cattle, deer, sheep, goat, horses, cats, dogs, and exotic species. The species within MAC are divided into the following subspecies according to taxonomical classification: *MAA*, the etiological agent of avian tuberculosis; *M. avium* spp. *paratuberculosis*, the etiological agent of Johne’s disease; *M. avium* spp. *silvaticum*, previously called “wood pigeon *Mycobacterium*”; *M. avium* spp. *hominissuis*, frequently isolated from pig and human and *M. intracellulare*, a closely related pathogen of birds with a lower prevalence (2, 6, 10). Although the subspecies of MAC differ greatly in their host range and growth potential, it has been reported that tuberculous lesions caused by *MAA, M. avium* spp. *paratuberculosis, M. avium* spp. *hominissuis*, and *M. avium* spp. *silvaticum* are indistinguishable (11–15). In addition, coinfection with different members of M. tuberculosis complex and *M. avium* subspecies or with other mycobacterial species combinations is not rare in animal and human hosts (16, 17). Accurate identification and discrimination of mycobacterial species and subspecies is essential to determine their significance, pathogenicity, diagnosis, epidemiology, and most beneficial control program (18–20).

The identification and preservation of microbial strains is an important strategic resource for disease epidemiology research and disease prevention. Since the first report of avian tuberculosis in 1978 in China, there have been additional reports about the disease (7). As known, avian tuberculosis is sporadic and has a low incidence. Recent years, there were reports about incidence of avian disease in the poultry farm, but not large scale occurrences of disease (7, 21, 22). Isolation and identification of *MAA* strains from pathological materials could provide abundant resources for avian tuberculosis research. Although more attention has recently been paid to avian tuberculosis research, fewer *MAA* strains have been isolated and identified in recent years because of the high biological safety risks and low economic value of *MAA* strains in China. There were almost no reports regarding the isolation of *MAA* strains from diseased species, and no systematic studies were carried out on the characteristics of *MAA* strains.

In this work, we first isolated an *MAA* strain from the typical tubercular nodules of a fowl with possible avian tuberculosis symptoms. We then systematically identified the isolated strain *via* a series of assays and further investigated the virulence and potency of the identified strain.

## Materials and Methods

### Ethics Statement

The present study was approved by the Laboratory Animal Ethics Committee of China Institute of Veterinary Drug Control and was also approved by the Ministry of Agriculture and the Bureau of Animal Husbandry. The experiments were performed in compliance with the “Regulations of the People’s Republic of China on the Administration of Experimental Animals” and the “Guidelines for the ethical review of experimental animal welfare in Beijing.”

*Mycobacterium avium* spp. *avium* strains were cultured in an air-conditioned, air filtered, biosafety level III facility. The experimental use of serum samples, including sample collection, handling, testing, and personal protection, complied with the General Requirements for Laboratory Biological Safety of China, GB19489 (2008).

### MAA Strains

*Mycobacterium avium* spp. *avium* strains CVCC275, CVCC276, and CVCC277 were the virulent reference strains for serotype 1, serotype 2, and serotype 3, respectively. *MAA* strain CVCC68201 was the virulent strain for avian tuberculin purified protein derivative (PPD) production and inspection (23).

### Tissue Origin and Examination

One fowl showing the clinical signs (swollen joints, lameness, emaciation, tubercle formation under the skin, and granulomas in the conjunctival sac) of avian tuberculosis and poor health was transferred to the avian diseases section, Shandong Agricultural University, College of Animal Science and Veterinary Medicine. The fowl was euthanized and underwent a necropsy examination, and tissue containing typical tubercles was collected in 50 mL screw cap containers, packed in dry ice chambers, and then sent to the China Institute of Veterinary Drug Control.

After thawing the tissue samples, portions were formalin fixed, embedded in paraffin blocks, and stained by the Ziehl-Neelsen (Z-N) technique to detect *Mycobacteria* (24). An additional tissue sample was fixed in neutral-buffered formalin and embedded in paraffin wax. Thereafter, the sample was sectioned, stained and transported to perform histopathological examination.

### Mycobacterial Isolation

Approximately 1 g of tissues were pooled into a test tube containing 1 mL of sterile PBS and homogenized for 1 min. Two hundred microliters of the mixture was inoculated onto two slopes of Petragnani medium (25). The inoculated slopes were incubated at 37°C for 4 weeks. Colony morphology was visualized with the naked eye.

### Grown Colony Staining

The grown colony on the slope was checked by Gram staining and Z-N acid-fast staining (24, 26). The studies were carried out using a Gram-staining kit and Z-N staining kit (Land Bridge Technology Co., Ltd., Beijing, China) according to the manufacturer’s instructions. The colony, confirmed to contain Gram-positive bacteria and acid-fast bacteria (AFB), was streaked out for further study.

### Biochemical Identification of the AFB Isolates

Biochemical identification of the AFB isolates was based on multiple tests, which included the p-nitrobenzoic acid (PNB) growth assay, niacin production, Tween-80 hydrolysis, heat stable catalase activity, and nitrate reductase activity (27, 28).

#### PNB Growth Assay

The AFB isolates were screened by a growth test on a slope of Petragnani medium containing 500 µg/mL PNB (29). The inoculated slope was incubated at 37°C for 4 weeks, and then growth conditions were visualized.

#### Niacin Production Assay

The AFB isolates were washed off from the slope of Petragnani medium. Next, 0.9 mL of bacterial suspension was added to 0.2 mL of 3% benzidine ethanol, followed by the addition of 0.2 mL of 10% hydrogen bromide. The color reaction and precipitation reaction were observed.

#### Tween-80 Hydrolysis Assay

The Neutral Red/Tween-80 solution was prepared by adding 0.5 mL of 0.8% Neutral Red to 100 mL of 0.5% Tween-80/PBS solution (pH 7.0), followed by sterilization at 116°C for 20 min. The AFB isolates were washed off from the slope and resuspended to a final concentration of 10 mg/mL with PBS. Then, 0.5 mL of bacterial suspension was added to 2 mL Neutral Red/Tween-80 solution and incubated at 37°C for 10 days. The color change of the Neutral Red/Tween-80 solution was observed daily.

#### Heat Stable Catalase Assay

First, 0.5 mL of the bacterial suspension prepared above (10 mg/mL) was added to a test tube and incubated at 68°C for 20 min. Then, 0.5 mL of 30% H_2_O_2_ in 10% Tween-80 (pH 7.0) was added to the bacterial culture. The evolution of O_2_ gas caused frothing indicative of catalase activity, whereas the absence of O_2_ gas bubbles demonstrated a loss of enzymatic activity.

#### Nitrate Reductase Assay

Nitrate reduction was performed by the classical procedure with liquid reagent. First, 10 mM NaNO_3_/PBS solution (pH 7) was prepared and sterilized at 116°C for 20 min. Next, 0.5 mL of the bacterial suspension prepared above (10 mg/mL) was added to 2 mL of NaNO_3_/PBS solution. The mixture was incubated at 37°C for 2 h, followed by the addition of 100 µL of 18% HCl and 100 µL of 0.2% sulfanilamide solution and later by 100 µL of 0.1% naphthylethylenediamine solution. The system was stored at 2–8°C for 2 weeks, and the color reaction was visualized.

### Genetic Identification of the AFB Isolates

The AFB isolates were examined by PCR for the detection of DnaJ, IS1245, and IS901 gene fragments (30). The colony on Petragnani medium was washed off from the slope and washed with PBS. After heat inactivation at 60°C for 2 h, genomic DNA was extracted using a genomic DNA purification kit (Promega, Madison, WI, USA). Primers were designed for amplification of DnaJ, IS*1245*, and IS*901* fragments (Table 1) (30). Multiplex PCR was programmed as 1 cycle at 96°C for 2 min, 35 cycles at 96°C for 10 s, 58°C for 10 s, 72°C for 1 min, and 1 cycle at 72°C for 2 min. The PCR products were visualized by DNA gel electrophoresis.

**Table 1:** The PCR primers used to identify the acid-fast bacteria isolates.

Gene	Sequence of primer	Length of PCR product (bp)
DnaJ	5′-GACTTCTACAAGGAGCTGGG-3′	140
	5′-GAGACCGCCTTGAATCGTTC-3′	
IS*1245*	5′-GAGTTGACCGCGTTCATCG-3′	385
	5′-CGTCGAGGAAGACATACGG-3′	
IS*901*	5′-GGATTGCTAACCACGTGGTG-3′	577
	5′-GCGAGTTGCTTGATGAGCG-3′	

### Serological Identification of the AFB Isolates

The serotype of the AFB isolates was identified by the serum agglutination method as described below (31). The antigens of serotype 1, 2, and 3 were prepared from *MAA* reference strains CVCC275, CVCC276, and CVCC277, respectively. The AFB isolates, as well as the three strains, were cultured on Lowenstein-Jensen solid medium at 37°C for 18 days. The bacteria were washed off using 200 mL of sterile PBS containing sterile glass beads and inactivated at 37°C for 7 days with shaking up to four times a day. The mixture was centrifuged to remove the supernatant. After washing three times, the bacterial pellet was suspended in 0.4% sodium citrate/PBS to a concentration of approximately 10^10^ CFU/mL. The suspended bacteria were used as antigen for the serum agglutination assay.

The AFB isolates were cultured on Petragnani medium at 37°C for 4 weeks. The bacterial culture was washed off using sterile PBS containing sterile glass beads and centrifuged at 10,000 rpm for 5 min to remove the supernatant. The bacterial pellet was weighed and resuspended in sterile PBS to a concentration of 0.1 mg/mL. Three 8-week-old SPF chickens were inoculated intravenously with 0.2 mL of the above bacterial suspension, with the same amount of PBS as a control. After 60 days, sera were collected from the wing vein for the serum agglutination assay.

For the serum agglutination assay, one drop of serum from the wing vein and one drop of bacterial suspension were mixed on a slide. After 1 min, the results were graded as negative (−), weakly positive (+), positive (++), and strongly positive (+++), according to agglutination degree as no agglutination (−), less than 25% agglutination (+), approximately 50% agglutination (++), and more than 75% agglutination (+++), respectively.

### Virulence

The AFB isolates were cultured on Petragnani medium at 37°C for 4 weeks. The bacterial culture was washed off using sterile PBS and centrifuged at 10,000 rpm for 5 min to remove the supernatant. The bacterial pellet was weighed. The virulence of the AFB isolates in rabbits and chickens was studied according to the “The People’s Republic of China veterinary biological product regulation” (25).

For virulence in rabbits, the above bacterial pellet was resuspended in sterile PBS to a final concentration of 0.5, 1, 2, or 4 mg/mL. Six healthy rabbits at 2 kg of body weight (Albino, Vital River) were inoculated intravenously in the ear with 1 mL of the bacterial suspension, while the same amount of PBS was used as a control. The inoculated rabbits were observed for 30 days, and survival was recorded.

For virulence in chickens, the above bacterial pellet was resuspended in sterile PBS to a final concentration of 0.5, 1.25, 2.5, or 5 mg/mL. Five 8-week-old SPF chickens (Vital River) were inoculated intravenously with 0.2 mL of the bacterial suspension, while the same amount of PBS was used as a control. The inoculated rabbits were observed for 60 days, and survival was recorded.

### Potency and Specificity

#### Preparation of PPD of Avian Tuberculin

Avian tuberculin was prepared according to the rules for tuberculin production and inspection (25), as described briefly below. The AFB isolates were streaked out from the Petragnani slope medium to 200 mL of Sauton liquid medium and grown at 37°C for 40–45 days to prepare the seed strain. The seed strain was inoculated into 1,000 mL of Sauton liquid medium and grown at 37°C for 90 days. The culture was inactivated at 121°C for 30 min and filtered through a hollow fiber membrane to remove the bacteria. The filtrate was mixed with 40% (w/v) TCA solution to a final concentration of 4%. The mixture was incubated overnight at 2–8°C. The pellets were collected and washed three times with 1% TCA solution. The pellets were centrifuged at 4°C, 5,000 rpm for 30 min, and the pellet was dried and weighed. The pellet was dissolved in 1 M NaOH, adjusted to pH 7.4 using 1 M HCl, and filtered through sterilizing Chua’s filter. The final product was the avian tuberculin PPD for further testing, referred to as PPD-AFB. The avian tuberculin PPD from CVCC68201 (PPD-CVCC68201) was prepared using the same procedure.

#### Preparation of Allergen and Sensitization of Guinea Pigs

*Mycobacterium avium* spp. *avium* strain CVCC68201 was cultured at 37°C for 20 days. The bacteria were scraped from the slope, weighed, dissolved in sterile PBS, and inactivated at 121°C for 30 min. The bacterial suspension was mixed with Fruend’s incomplete adjuvant to prepare an emulsion at a concentration of 40 mg/mL bacteria. The emulsion was subpackaged and inactivated at 80°C water bath for 2 h, and the emulsion was used as the allergen. Sixteen guinea pigs at 400 g of body weight (Hartly, Vital River) were sensitized with 0.5 mL of allergen by deep intramuscular injection in the inner thigh and maintained for 35 days under specific pathogen-free conditions. After the sensitization period, guinea pigs were shaved in a 3 cm^2^ area on the hip, at the opposite side of allergen injection. The standard avian tuberculin PPD (PPD-S, IVDC) was diluted 100-fold, and 0.1 mL diluted standard avian tuberculin PPD was injected into the shaved area of the guinea pigs. The diameter of reactions on the skin was measured after 24 h, and the guinea pig was positive for sensitization if the area of skin thickness was over 1 cm^2^.

#### Potency Calibration and Specificity Test of Avian Tuberculin PPD

The potency calibration of PPD was carried out according to the relevant rules (24–26, 32). For the potency calibration, the PPD-AFB was diluted to two concentrations of 0.1 and 0.25 dose/mL. Additionally, the PPD-S and PPD-CVCC68201 were diluted to two concentrations of 100 and 250 IU/mL. The shaved area of the sensitized guinea pigs was divided into two equal blocks. Each block contained three randomized injection sites resulting in six total injections, with a replicated injection of each PPD concentration in each guinea pig. Guinea pigs were injected intradermally with a volume of 0.1 mL of designated PPDs. A total of 12 guinea pigs (6 guinea pigs for each PPD concentration) were used for PPD inoculation. An additional 4 guinea pigs were used as negative controls. No adverse effect resulted from the procedures used, and the animals were monitored daily by the animal care staff. After 24 h, the skin thickness was measured using Vernier calipers.

The specificity of PPD testing was performed as described below (25, 33). Six guinea pigs at 400 g of body weight (Hartly, Vital River) were shaved in an area (3 cm × 9 cm) on both sides of the chest. PPD-AFB, PPD-S, and PPD-CVCC68201 were diluted to 2 × 10^4^ IU/mL. Similar to the potency test, the guinea pigs were injected intradermally with one volume of 0.1 mL of designed PPDs with replicated injections of each PPD. After 24 h, the skin inflammatory reaction was observed.

### Statistical Analysis

The statistical package SPSS and one-way ANOVA method were used for data analyses. The results were expressed as the mean ± SD. The *p*-values were considered significant at (*p<0.05).

## Results

### Identification of the Pathological Tissue

**Figure 1:**
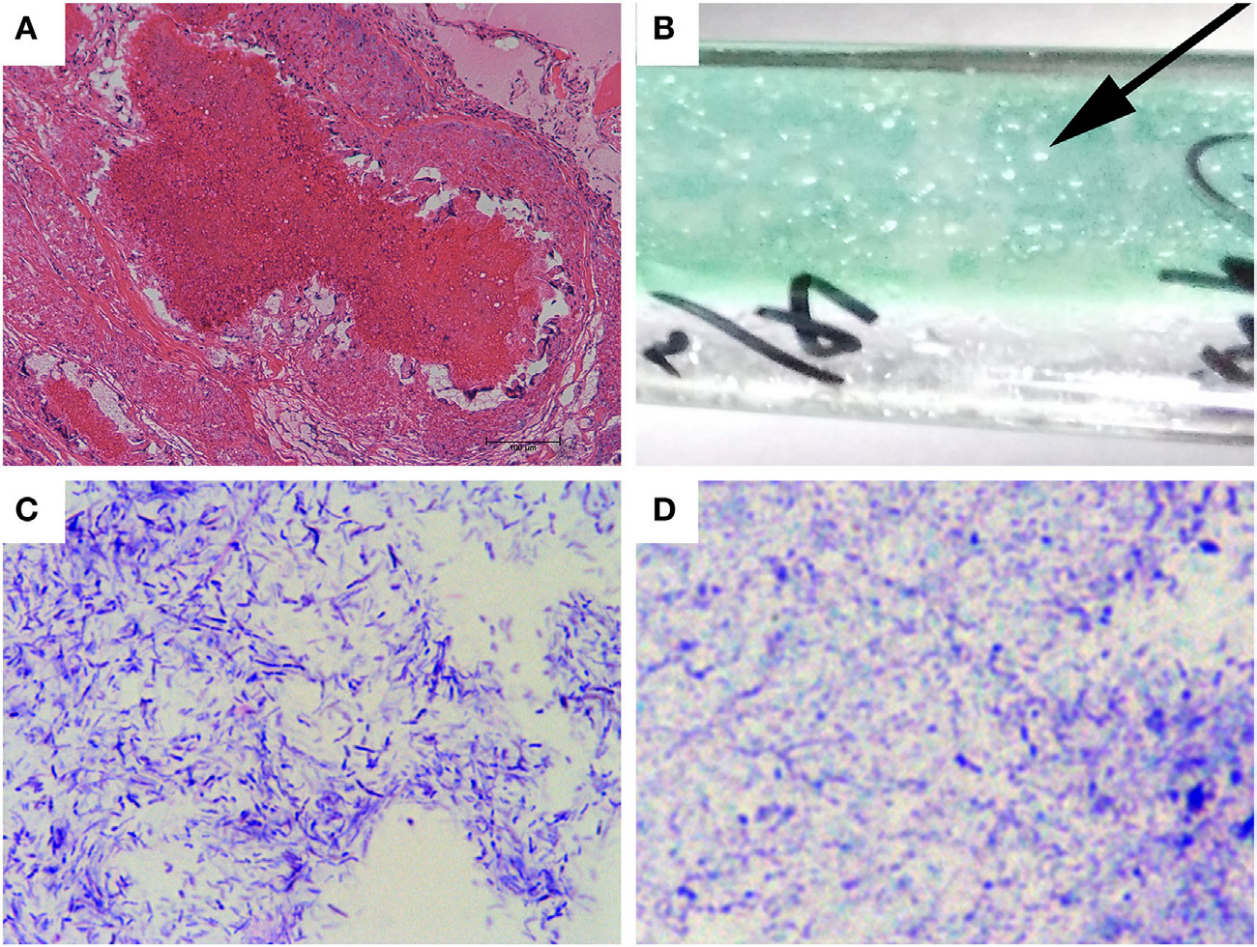
Tubercle staining and mycobacterial isolation. **(A)** Histopathological staining of the pathological tubercle from the diseased fowl. Bar, 100 µm. **(B)** Growth of isolated mycobacteria colonies from the tissue homogenate on a slope of Petragnani medium. The arrow indicates a typical clone. **(C,D)** Photographies of the mycobacterial colonies after Gram staining **(C)** and Z-N acid-fast staining [**(D)**, 1,000×].

The pathological tubercle contained a yellow-white cheese-like substance with a coated envelope after cutting. After smearing and Z-N staining, a large number of mycobacteria were observed (data not shown). With further histopathological examination of the tubercle, caseous necrosis appeared in the center of the tubercle with a large number of lymphoid cells, epithelioid cells, and Langerhans multinucleated giant cells (Figure 1A). This was a typical avian tuberculosis symptom.

On the slopes of the Petragnani medium inoculated with homogenized tissue, transparent, round, thick, drip-like colonies were observed (Figure 1B, arrow) after incubation at 37°C for 4 weeks. The colonies did not have a yellowish-white, cream like appearance (24, 25, 34). While streaking, the colonies had a threadlike filament (data not shown). The colonies were further tested by Gram staining and Z-N staining. As shown in Figure 1C, the colonies were Gram-positive, bluish-purple staining, and contained straight or curved rod-shaped bacteria. Additionally, the grown colonies were Z-N staining positive with red staining and a length of 1–3 µm (Figure 1D). The above results indicated that the colonies were AFB, i.e., mycobacteria.

### Biochemical Characterization of AFB Isolates

Five different biochemical tests, including resistance to PNB, niacin production, Tween-80 hydrolysis, heat stable catalase and nitrate production, were applied to the AFB isolates (Table 2). The AFB isolates showed positive results for resistance to PNB. For the niacin production test, a clear pellet, not a peach red pellet was observed, indicating a negative result. The test for Tween-80 hydrolysis was negative, indicated by no color change of from neutral red. The heat stable catalase activity was negative, as no bubble was generated in the reaction solution. The reaction system was very light pink, suggesting the absence of nitrate reductase in the AFB isolates. From the above biochemical tests, the AFB isolates most closely resemble*MAA* in their enzymatic activities.

**Table 2:** Distinguishing characteristics of the acid-fast bacteria (AFB) isolates.

Characteristics	Resistant to p-nitrobenzoic acid	Niacin production	Tween-80 hydrolysis	Catalase at 68°C	Nitrate reduction
AFB isolates	+	−	−	−	−

### Genetic Characterization of AFB Isolates

**Figure 2:**
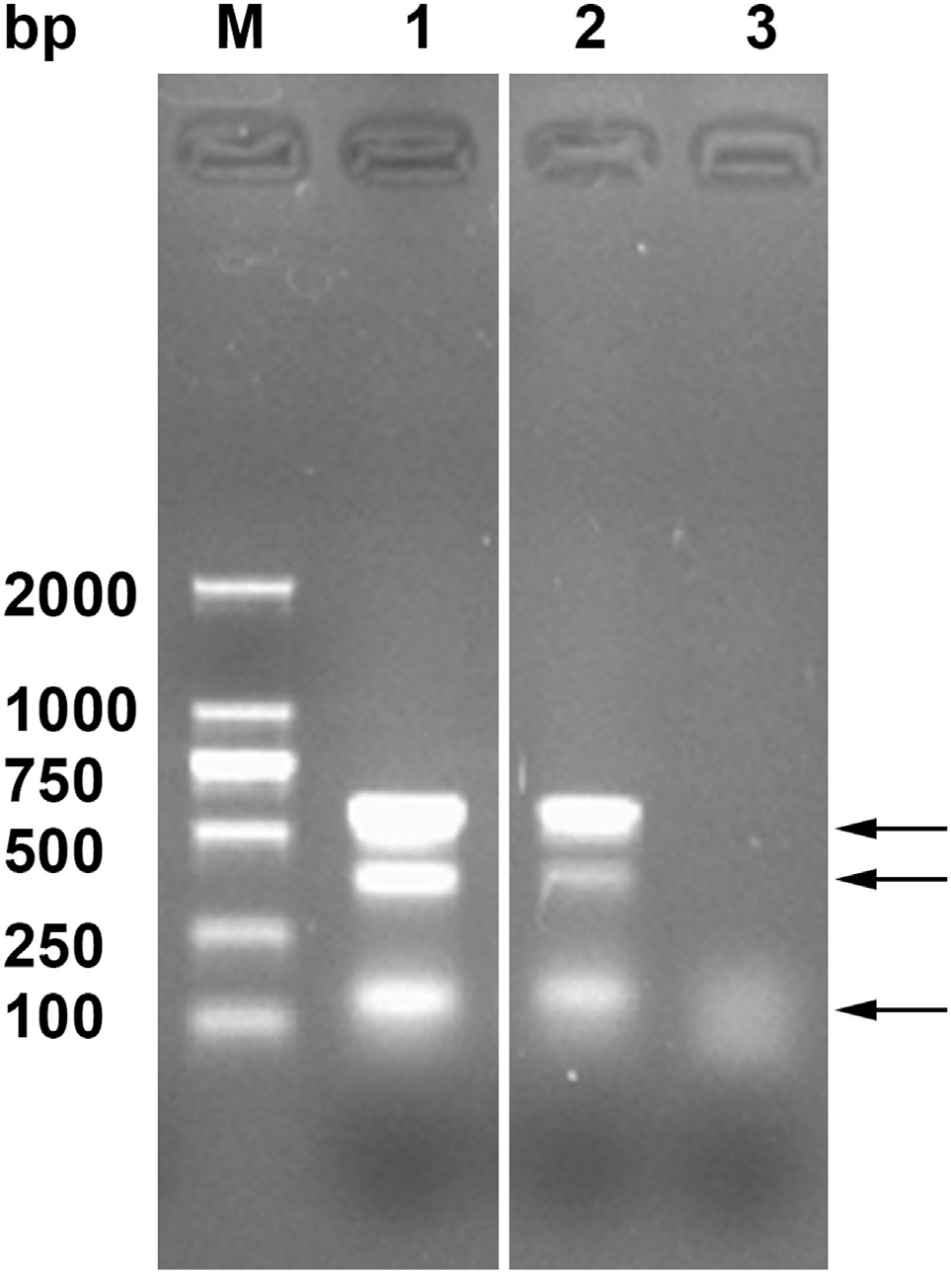
Genetic identification of the acid-fast bacteria (AFB) isolates by Multiplex PCR. Lane M, DNA ladder, Lane 1, AFB isolates, Lane 2, CVCC68201, and Lane 3, PBS. The arrows indicate the three typical amplified bands of DnaJ gene, *IS1245*, and *IS901*.

Using multiplex PCR, the AFB isolates and the CVCC6801 strain produced three bands (140, 385, and 577 bp) corresponding to the DnaJ gene, IS*1245*, and IS*901* (Figure 2). As the isolates were *IS901*+, *IS1245*+, and dnaJ+ (30, 35), the isolates were of the *MAA* strain.

### Serological Characterization of AFB Isolates

The *MAA* strain has three serotypes: serotype 1, serotype 2, and serotype 3 (2). Using the serum agglutination method, the antigen prepared from AFB isolates showed a strong agglutination reaction to the serum from chickens infected with the CVCC275 strain but no agglutination reaction to the serum from chickens infected with the CVCC276 and CVCC277 strains (Table 3). The *MAA* strains CVCC275, CVCC276, and CVCC277 were the reference strains for serotype 1, serotype 2, and serotype 3, respectively. The results suggested that the AFB isolates were an *MAA* strain of serotype 1.

**Table 3:** Serotype identification of the acid-fast bacteria (AFB) isolates.

	Antigen prepared from		
	CVCC275	CVCC276	CVCC277
Sera^a^ from the AFB isolates inoculated chicken	+++	−	−

*^a^The sera from three inoculated chicken was all tested and showed same result*.

### Virulence of the AFB Isolates

**Figure 3:**
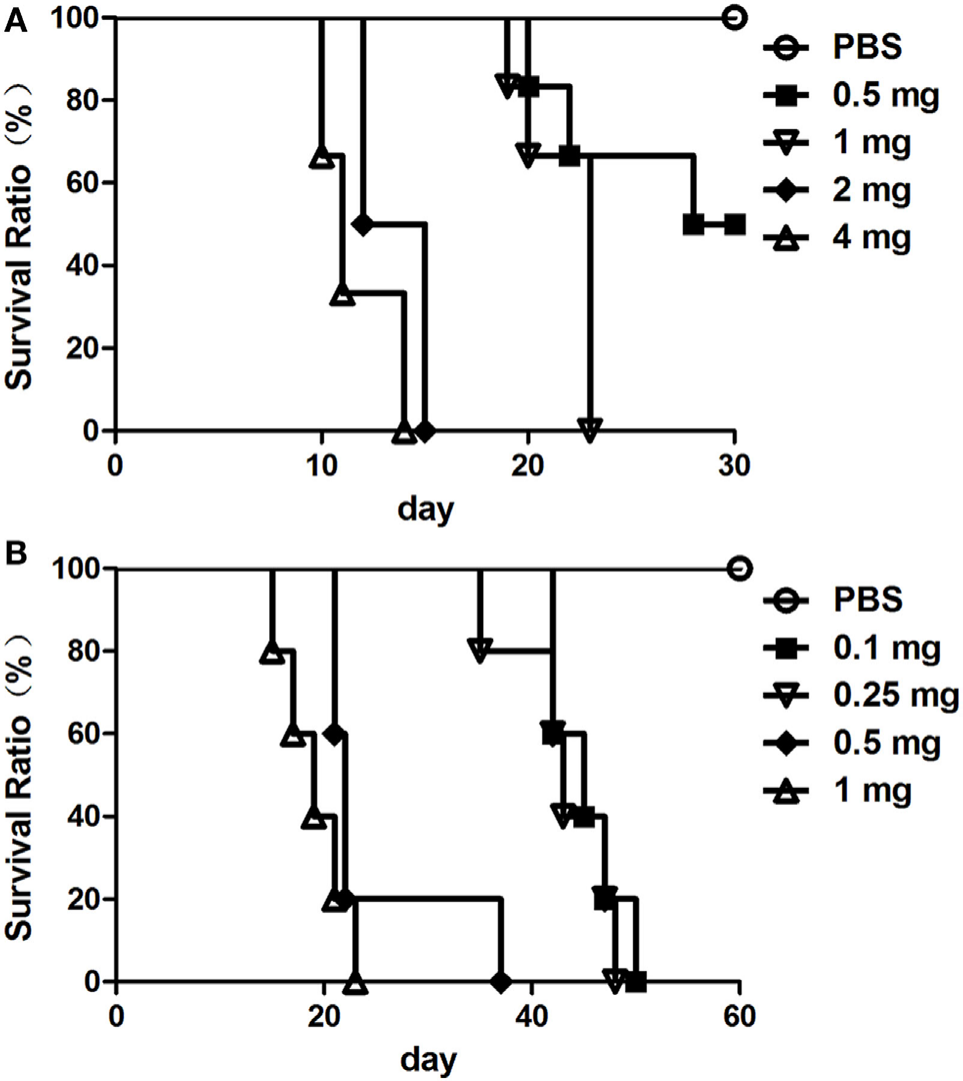
The virulence of the acid-fast bacteria (AFB) isolates. Five groups of healthy rabbit **(A)** and chicken **(B)** was inoculated with different amounts of *Mycobacterium avium* spp. *avium* bacteria. The virulence of the AFB isolates is represented by the survival ratio of rabbits **(A)** and chickens **(B)**.

To evaluate the virulence of the AFB isolates, mortality in rabbits and chickens was recorded during the observation period post-injection of the prepared bacteria. As shown in Figure 3A, the six uninfected rabbits remained healthy and alive until the 30th day of the observation period. Among the six rabbits injected with 0.5 mg of bacteria, three of the rabbits died, and three remained alive until the end of the observation period. For the remaining three groups of rabbits inoculated with 1, 2, and 4 mg of bacteria, all rabbits died during the observation period. Moreover, the rabbits died at earlier dates when inoculated with higher doses of bacteria.

As shown in Figure 3B, the five uninfected chickens remained healthy and alive until the 60th day of the observation period. All of the chickens in the groups injected with different doses of bacteria, from 0.1 to 1.0 mg, died during the 60 days observation period. Additionally, the chickens died earlier when given a higher inoculation dose of bacteria.

According to the definition of *MAA* virulence (25), the results indicated that the AFB isolates were *MAA* strain with strong virulence.

### Potency and Specificity of the PPD Prepared from AFB Isolates

**Figure 4:**
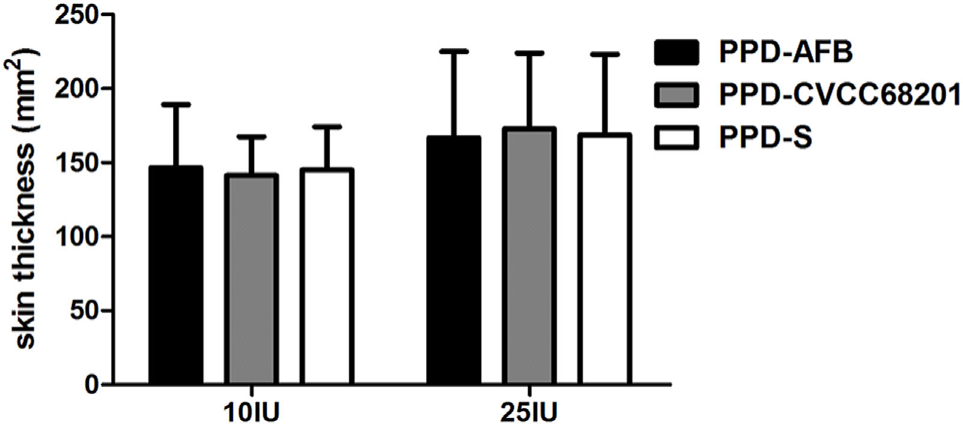
The potency of the acid-fast bacteria (AFB) isolates. The purified protein derivative (PPD) was prepared from the AFB isolates and was calibrated by traditional PPD skin test. The potency is represented by the area of skin thickness after PPD injection on sensitized guinea pigs.

The potency of the AFB isolates was evaluated by calibrating the prepared avian tuberculin PPD from the AFB isolates. As shown in Figure 4, PPD-AFB, PPD-S and PPD-CVCC68201 showed no significant difference when analyzed for indurations (skin thickness) in sensitized guinea pigs. Thus, the PPD prepared from the AFB isolates had a similar potency to the standard PPD and the PPD from the reference *MAA* strain, and the AFB isolates had a similar potency to the reference *MAA* strain.

The specificity of the AFB isolates was analyzed by the skin test in unsensitized guinea pigs with PPD-AFB, PPD-CVCC68201, and PPD-S. Similar to PPD-CVCC68201 and PPD-S, PPD-AFB could not induce a skin inflammatory reaction (Table 4). Thus, the PPD prepared from the AFB isolates was specific for PPD prepared from the *MAA* strains.

**Table 4:** Specificity of the p-nitrobenzoic acid (PPD) prepared from the acid-fast bacteria isolates.

PPD	Testing-PPD	PPD-S	PPD-CVCC68201
Skin inflammatory reaction	−	−	−

## Discussion

In this work, we isolated an *MAA* strain from caseous tubercular nodules of a fowl with avian tuberculosis. The isolated strain was a typical *MAA* strain of serotype 1, genotype IS*901*+ and IS*1245*+ according to biochemical, serological and genetic identification. The identified *MAA* strain was a virulent strain and showed similar PPD potency as the reference *MAA* strain. Interestingly, our study revealed that this isolated *MAA* strain showed a novel colony appearance not previously reported.

Diagnosis of avian tuberculosis is frequently based on clinical symptoms, postmortem gross lesions and Z-N staining of the smear. However, Z-N staining for demonstrating acid-fast bacilli is limited because of its low sensitivity and detection rate (36). Serological diagnosis method such as hemagglutination, complement fixation, ELISA, and Western-blot analysis might be most useful in late-stage infections based on the process of mycobacteria evading the host immune system. Also the request of specific antigen might limit the board application of these methods (37). The PCR approaches, including PCR, multiplex PCR, PCR restriction fragment length polymorphism (RFLP), and mycobacterial interspersed repetitive units and variable number tandem repeats (MIRU-VNTR) typing are also capable of specifically detecting DNA fragments, thus acting as a diagnostic procedure (30, 35, 38, 39). However, PCR products can have false positive results because of contamination (40). The PPD skin test is the only method recommended by OIE, but the result reading is affected by the dose and injection manner (24). Comparing everything, the gold standard technique for avian tuberculosis is culturing from diseased tissue for isolation, genotypic and phenotypic characterization of the suspect pathogen (2). This method is reliable, but it requires more time and high quality culture medium.

For the conventional culturing technique of the *MAA* strain, it is important to use the proper selective medium (41). It was reported that *M. avium* grows best on media such as Lowenstein-Jensen medium, Herrold’s medium and Middlebrook medium (6), which were also recommended by the World Organization for Animal Health as the culturing medium of *MAA* (24). In this study, we used Petragnani medium to isolate the *MAA* strain from the tubercle (25, 26). The Petragnani medium was reported to have similar colony yielding ratio as Lowenstein-Jensen medium for Mycobacterium tuberculosis (42). As known, culturing of mycobacteria is time-consuming to complete, as it takes at least 4–8 weeks for visible colonies to appear (5, 37). For *MAA* strain, the culture should be incubated for at least 12 weeks using Lowenstein-Jensen medium, Herrold’s medium and Middlebrook medium (43). It took only 4 weeks to observe transparent, round, thick, drip-like colonies on Petragnani medium (Figure 1B). Thus, culturing Petragnani medium for isolation of *MAA* strain was time-saving and offered greater efficiency for avian diagnosis.

The traditional method for species and subspecies typing of *Mycobacterium* was according to growth on distinct medium and morphology of the grown colony. The *MAA* strain usually had yellowish-white, round, and cream-like colonies (25) (Insights into the mycobacterium infection). In our study, the isolated *MAA* strain had a novel colony appearance, as round, thick, drip-like colonies (Figure 1B). *MAA* forms colonies of different morphologies, including smooth transparent, smooth opaque, and rough, which might influence pathogenicity, virulence, drug susceptibility and macrophage survival (44–46). Here, this isolated *MAA* strain had similar virulence and PPD potency compared to the *MAA* reference strain CVCC68201 (Figures 3 and 4). This finding might indicate that this isolated *MAA* strain might be of a novel subtype and have differences in some unknown phenotype. However, the traditional method was limited in use because of the high biosafety requirement, and it was more time-consuming and required more complicated techniques. Genetic approaches would applied for further subtyping of this newly isolated *MAA* strain, as RFLP and DNA sequencing of distinct gene cluster for subtyping of diverse *MAA* isolates (47, 48), short sequence repeats sequencing and MIRU-VNTR typing for subtyping of *M. avium* spp. *paratuberculosis* (49–51). For example, IS901 RFLP typing was used to discriminate *MAA* field isolates (52, 53). As reported, a cluster of genes were proved to be related to colony morphology, such as MAP1156 (diacyglycerol O-acyltransferase), MAP 1152 (PPE protein), and Lsr2 etc. in *M. avium* spp. *paratuberculosis* (54), MAV_4334 (nitroreductase family protein), MAV_5106 (phosphoenolpyruvate carboxykinase), MAV_1778 (GTP-binding protein LepA), etc. in *M. avium* spp. *hominissuis* (55). In addition, glycopeptidolipids and its biosynthesis were reported to be related to the surface properties and colony morphology of mycobacteria (56–58). DNA sequencing of these gene clusters might give a clue for further subtyping of this isolated *MAA* strain with a round, thick, drip like colony appearance (Figure 1B).

Virulence is a reflection of pathogenicity of the bacterial pathogen. The virulence of *MAA* is affected by various factors and could change with different hosts and environments (59). The extent of intracellular replication in cell cultures, guinea pigs and mice is widely used as a measure for mycobacterial virulence (60, 61). Additionally, the mortality of animals inoculated with mycobacteria is also used as an indicator of virulence (25, 62). The definition of the *MAA* virulent strain is that inoculation of 0.1–1.0 mg bacteria would lead to the death of chickens or that 1 mg of bacteria would lead to the death of rabbits (23, 25). The isolated *MAA* strain here was proven to have strong virulence (Figure 3). Compared to intracellular bacterial replication or macrophage infection (59, 63), it was more intuitive to use mortality as an indicator of virulence, especially for a bacterial pathogen of strong virulence.

In conclusion, in this study, we isolated and identified a strain of *M. avium* spp. *avium*. The virulence and the derivative PPD of the strain were also characterized. Our work provided a novel reference strain for the intensive study of avian tuberculosis. This work not only enriched strain resources for *M. avium* spp. *avium* but also provided a realistic candidate strain for further development of *M. avium* spp. *avium* vaccines.

## Ethics Statement

The present study was approved by the Laboratory Animal Ethics Committee of China Institute of Veterinary Drug Control and was also approved by the Ministry of Agriculture and the Bureau of Animal Husbandry. The experiments were performed in compliance with the “Regulations of the People’s Republic of China on the Administration of Experimental Animals” and the “Guidelines for the ethical review of experimental animal welfare in Beijing.” *MAA* strains were cultured in an air-conditioned, air filtered, biosafety level III facility. The experimental use of serum samples, including sample collection, handling, testing, and personal protection, complied with the General Requirements for Laboratory Biological Safety of China, GB19489 (2008).

## Author Contributions

LZ conceived and designed the experiments and wrote the whole manuscript. YP, JY, TW, ZB, and HZ provided assistance of the experiments. YQ revised the manuscript. JD supervised the experiments and revised the manuscript.

## Conflict of Interest Statement

The authors declare that the research was conducted in the absence of any commercial or financial relationships that could be construed as a potential conflict of interest.

## References

[1] Marco I Domingo M Lavin S. *Mycobacterium* infection in a captive-reared capercaillie (*Tetrao urogallus*). Avian Dis (2000) 44:227–30. 10.2307/1592531 10737668

[2] Saif YM Fadly AM Diseases of Poultry. Ames, Iowa: Blackwell Pub (2008).

[3] Slany M Ulmann V Slana I. Avian mycobacteriosis: still existing threat to humans. Biomed Res Int (2016) 2016:4387461. 10.1155/2016/4387461 27556033 PMC4983314

[4] Martin G Schimmel D [*Mycobacterium avium* infections in poultry – a risk for human health or not?]. Dtsch Tierarztl Wochenschr (2000) 107:53–8. 10743334

[5] Dhama K Mahendran M Tomar S Avian tuberculosis: an overview. Poul Punch (2007) 24:38–52.

[6] Dhama K Mahendran M Tiwari R Dayal Singh S Kumar D Singh S Tuberculosis in birds: insights into the *Mycobacterium avium* infections. Vet Med Int (2011) 2011:712369. 10.4061/2011/712369 21776352 PMC3135220

[7] Wang X Overview of avian tuberculosis in China. Guide to Chin Poul (2001) 18:20–2.

[8] Buur J Saggese MD. Taking a rational approach in the treatment of avian mycobacteriosis. Vet Clin North Am Exot Anim Pract (2012) 15:57–70,vi. 10.1016/j.cvex.2011.12.001 22244113

[9] Saggese MD Tizard I Gray P Phalen DN. Evaluation of multidrug therapy with azithromycin, rifampin, and ethambutol for the treatment of *Mycobacterium avium* subsp *avium* in ring-neck Doves (*Streptopelia risoria*): an uncontrolled clinical study. J Avian Med Surg (2014) 28:280–9. 10.1647/2012-067R1 25843465

[10] Rindi L Garzelli C. Genetic diversity and phylogeny of *Mycobacterium avium*. Infect Genet Evol (2014) 21:375–83. 10.1016/j.meegid.2013.12.007 24345519

[11] Mackintosh CG De Lisle GW Collins DM Griffin JF. Mycobacterial diseases of deer. N Z Vet J (2004) 52:163–74. 10.1080/00480169.2004.36424 15726126

[12] Turenne CY Collins DM Alexander DC Behr MA. *Mycobacterium avium* subsp. *paratuberculosis* and *M. avium* subsp. *avium* are independently evolved pathogenic clones of a much broader group of *M. avium* organisms. J Bacteriol (2008) 190:2479–87. 10.1128/JB.01691-07 18245284 PMC2293204

[13] Agdestein A Johansen TB Kolbjornsen O Jorgensen A Djonne B Olsen I. A comparative study of *Mycobacterium avium* subsp. avium and *Mycobacterium avium* subsp. *hominissuis* in experimentally infected pigs. BMC Vet Res (2012) 8:11. 10.1186/1746-6148-8-11 22284630 PMC3296603

[14] Ronai Z Csivincsik A Dan A Gyuranecz M. Molecular analysis and MIRU-VNTR typing of *Mycobacterium avium* subsp. avium, ‘*hominissuis*’ and silvaticum strains of veterinary origin. Infect Genet Evol (2016) 40:192–9. 10.1016/j.meegid.2016.03.004 26964909

[15] Arrazuria R Juste RA Elguezabal N. Mycobacterial infections in rabbits: from the wild to the laboratory. Transbound Emerg Dis (2017) 64:1045–58. 10.1111/tbed.12474 26799551

[16] Sharma K Sharma A Modi M Singh G Kaur H Varma S PCR detection of co-infection with *Mycobacterium tuberculosis* and *Mycobacterium avium* in AIDS patients with meningitis. J Med Microbiol (2012) 61:1789–91. 10.1099/jmm.0.045898-0 22935849

[17] Arrazuria R Sevilla IA Molina E Perez V Garrido JM Juste RA Detection of *Mycobacterium avium* subspecies in the gut associated lymphoid tissue of slaughtered rabbits. BMC Vet Res (2015) 11:130. 10.1186/s12917-015-0445-2 26063469 PMC4461944

[18] Chiers K Deschaght P De Baere T Dabrowski S Kotlowski R De Clercq D Isolation and identification of *Mycobacterium avium* subspecies silvaticum from a horse. Comp Immunol Microbiol Infect Dis (2012) 35:303–7. 10.1016/j.cimid.2012.01.011 22349520

[19] Ahlstrom C Barkema HW Stevenson K Zadoks RN Biek R Kao R Limitations of variable number of tandem repeat typing identified through whole genome sequencing of *Mycobacterium avium* subsp. *paratuberculosis* on a national and herd level. BMC Genomics (2015) 16:161. 10.1186/s12864-015-1387-6 25765045 PMC4356054

[20] Sevilla IA Molina E Elguezabal N Perez V Garrido JM Juste RA. Detection of mycobacteria, *Mycobacterium avium* subspecies, and *Mycobacterium tuberculosis* complex by a novel tetraplex real-time PCR assay. J Clin Microbiol (2015) 53:930–40. 10.1128/JCM.03168-14 25588660 PMC4390653

[21] Xu Q Zhou S Li Z Bi D Diagnosis of avian tuberculosis. Chin J Vet Sci (2001) 31:30–1. 10.3969/j.issn.1673-4696.2001.03.016

[22] Zhang Y Cai J Shen Y Diagnosis and therapy of avian tuberculosis. Poul Husbandry Dis Control (2002) 11:28–9.

[23] China Institute of Veterinary Drug Control, China Veterinary Culture Collection Center. CVCC Catalogue of Culture. Beijing, China: China Agricultural Science and Technology Publishing Company (2008).

[24] Office International Des Epizooties. OE Terrestrial Manual. Paris, France: Office International Des Epizooties (2014).

[25] Ministry of Agriculture of the People’s Republic of China. The People’s Republic of China Veterinary Biological Product Regulation. Beijing, China: Chemical Industry Press (2000). p. 164–6.

[26] General Administration of Quality Supervision, Inspection and Quarantine, People’s Republic of China Industry standard for import and export inspection and quarantine. In: Certification and Accreditation Administration of the Peoples Republic of China, editor. Quarantine Protocol for Animal Tuberculosis. Beijing: Standards Press of China (2011). p. 4–5.

[27] Levy-Frebault VV Portaels F. Proposed minimal standards for the genus *Mycobacterium* and for description of new slowly growing *Mycobacterium* species. Int J Syst Bacteriol (1992) 42:315–23. 10.1099/00207713-42-2-315 1581193

[28] Yang Z Fang H Human and Animal Pathogenic Bacteria. Shijiazhuang, Hebei: Hebei Science & Technology Press (2003).

[29] Sharma B Pal N Malhotra B Vyas L. Evaluation of a rapid differentiation test for *Mycobacterium tuberculosis* from other mycobacteria by selective inhibition with P-nitrobenzoic acid using MGIT 960. J Lab Physicians (2010) 2:89–92. 10.4103/0974-2727.72157 21346904 PMC3040081

[30] Moravkova M Hlozek P Beran V Pavlik I Preziuso S Cuteri V Strategy for the detection and differentiation of *Mycobacterium avium* species in isolates and heavily infected tissues. Res Vet Sci (2008) 85:257–64. 10.1016/j.rvsc.2007.10.006 18037461

[31] Chen X Mao K Luo Y Diagnosis of avian tuberculosis with the avian Tubercula plain agglutination test. Chin J Vet Drug (1999) 33:3–5.

[32] General Administration of Quality Supervision, Inspection and Quarantine, People’s Republic of China. National standards of the People’s Republic of China. In: National Technical Standardization Committee of Animal Inspection and Quarantine, editor. Diagnostic Techniques for Tuberculosis of Animal. Beijing: General Administration of Quality Supervision, Inspection and Quarantine (2002). p. 181–82.

[33] Haifeng X Testing method for the specificity of purified protein derivative. Chin J Vet Drug (1990) 24:15–8.

[34] Mahon CR Lehman DC Manuselis G Textbook of Diagnostic Microbiology. Maryland Heights, MO: Saunders (2011).

[35] Bartos M Hlozek P Svastova P Dvorska L Bull T Matlova L Identification of members of *Mycobacterium avium* species by Accu-Probes, serotyping, and single IS900, IS901, IS1245 and IS901-flanking region PCR with internal standards. J Microbiol Methods (2006) 64:333–45. 10.1016/j.mimet.2005.05.009 16061296

[36] Garg SK Tiwari RP Tiwari D Singh R Malhotra D Ramnani VK Diagnosis of tuberculosis: available technologies, limitations, and possibilities. J Clin Lab Anal (2003) 17:155–63. 10.1002/jcla.10086 12938143 PMC6807935

[37] Dahlhausen B Tovar DS Saggese MD. Diagnosis of mycobacterial infections in the exotic pet patient with emphasis on birds. Vet Clin North Am Exot Anim Pract (2012) 15:71–83,vi. 10.1016/j.cvex.2011.11.003 22244114

[38] Saito H Tomioka H Sato K Tasaka H Dawson DJ. Identification of various serovar strains of *Mycobacterium avium* complex by using DNA probes specific for *Mycobacterium avium* and *Mycobacterium intracellulare*. J Clin Microbiol (1990) 28:1694–7. 2203807 10.1128/jcm.28.8.1694-1697.1990 PMC268029

[39] Inagaki T Nishimori K Yagi T Ichikawa K Moriyama M Nakagawa T Comparison of a variable-number tandem-repeat (VNTR) method for typing *Mycobacterium avium* with mycobacterial interspersed repetitive-unit-VNTR and IS1245 restriction fragment length polymorphism typing. J Clin Microbiol (2009) 47:2156–64. 10.1128/JCM.02373-08 19403768 PMC2708485

[40] Ritelli M Amadori M Tagliabue S Pacciarini ML. Use of a macrophage cell line for rapid detection of *Mycobacterium bovis* in diagnostic samples. Vet Microbiol (2003) 94:105–20. 10.1016/S0378-1135(03)00080-4 12781479

[41] Tell LA Woods L Cromie RL Mycobacteriosis in birds. Rev Sci Tech (2001) 20:180–203. 10.20506/rst.20.1.1273 11288511

[42] Martin RS Sumarah RK Robart EM. Comparison of four culture media for the isolation of *Mycobacterium tuberculosis*: a 2-year study. J Clin Microbiol (1975) 2:438–40. 811686 10.1128/jcm.2.5.438-440.1975 PMC274204

[43] Mayahi M Mosavari N Esmaeilzadeh S Parvandar-Asadollahi K. Comparison of four different culture media for growth of *Mycobacterium avium* subsp. *avium* isolated from naturally infected lofts of domestic pigeons. Iran J Microbiol (2013) 5:379–82. 25848508 PMC4385164

[44] Belisle JT Brennan PJ Molecular basis of colony morphology in *Mycobacterium avium*. Res Microbiol (1994) 145:237–42. 10.1016/0923-2508(94)90024-8 7809478

[45] Reddy VM Luna-Herrera J Gangadharam PR. Pathobiological significance of colony morphology in *Mycobacterium avium* complex. Microb Pathog (1996) 21:97–109. 10.1006/mpat.1996.0046 8844653

[46] Cangelosi GA Palermo CO Bermudez LE. Phenotypic consequences of red-white colony type variation in *Mycobacterium avium*. Microbiology (2001) 147:527–33. 10.1099/00221287-147-3-527 11238960

[47] Krzywinska E Schorey JS. Characterization of genetic differences between *Mycobacterium avium* subsp. avium strains of diverse virulence with a focus on the glycopeptidolipid biosynthesis cluster. Vet Microbiol (2003) 91:249–64. 10.1016/S0378-1135(02)00292-4 12458173

[48] Parvandar-Asadollahi K Mosavari N Mayahi M. Genotyping of *Mycobacterium avium* subsp. *avium* isolates from naturally infected lofts of domestic pigeons in Ahvaz by IS901 RFLP. Iran J Microbiol (2015) 7:260–4. 26719782 PMC4695507

[49] Castellanos E Romero B Rodriguez S De Juan L Bezos J Mateos A Molecular characterization of *Mycobacterium avium* subspecies *paratuberculosis* types II and III isolates by a combination of MIRU-VNTR loci. Vet Microbiol (2010) 144:118–26. 10.1016/j.vetmic.2009.12.028 20116185

[50] Ricchi M Barbieri G Cammi G Garbarino CA Arrigoni N High-resolution melting for analysis of short sequence repeats in *Mycobacterium avium* subsp. *paratuberculosis*. FEMS Microbiol Lett (2011) 323:151–4. 10.1111/j.1574-6968.2011.02371.x 22092714

[51] Ricchi M Barbieri G Taddei R Belletti GL Carra E Cammi G Effectiveness of combination of mini-and microsatellite loci to sub-type *Mycobacterium avium* subsp. *paratuberculosis* Italian type C isolates. BMC Vet Res (2011) 7:54 10.1186/1746-6148-7-54 21929793 PMC3182896

[52] Dvorska L Bull TJ Bartos M Matlova L Svastova P Weston RT A standardised restriction fragment length polymorphism (RFLP) method for typing *Mycobacterium avium* isolates links IS901 with virulence for birds. J Microbiol Methods (2003) 55:11–27. 10.1016/S0167-7012(03)00092-7 14499991

[53] Moravkova M Lamka J Slany M Pavlik I. Genetic IS901 RFLP diversity among *Mycobacterium avium* subsp. *avium* isolates from four pheasant flocks. J Vet Sci (2013) 14:99–102. 10.4142/jvs.2013.14.1.99 23388436 PMC3615240

[54] Rathnaiah G Lamont EA Harris NB Fenton RJ Zinniel DK Liu X Generation and screening of a comprehensive *Mycobacterium avium* subsp. *paratuberculosis* transposon mutant bank. Front Cell Infect Microbiol (2014) 4:144. 10.3389/fcimb.2014.00144 25360421 PMC4197770

[55] Khattak FA Kumar A Kamal E Kunisch R Lewin A. Illegitimate recombination: an efficient method for random mutagenesis in *Mycobacterium avium* subsp. *hominissuis*. BMC Microbiol (2012) 12:204. 10.1186/1471-2180-12-204 22966811 PMC3511198

[56] Deshayes C Laval F Montrozier H Daffe M Etienne G Reyrat JM. A glycosyltransferase involved in biosynthesis of triglycosylated glycopeptidolipids in *Mycobacterium smegmatis*: impact on surface properties. J Bacteriol (2005) 187:7283–91. 10.1128/JB.187.21.7283-7291.2005 16237011 PMC1272997

[57] Johansen TB Agdestein A Olsen I Nilsen SF Holstad G Djonne B. Biofilm formation by *Mycobacterium avium* isolates originating from humans, swine and birds. BMC Microbiol (2009) 9:159. 10.1186/1471-2180-9-159 19660141 PMC2741467

[58] Mukherjee R Chatterji D. Glycopeptidolipids: immuno-modulators in greasy mycobacterial cell envelope. IUBMB Life (2012) 64:215–25. 10.1002/iub.602 22252955

[59] Viale MN Park KT Imperiale B Gioffre AK Colombatti Olivieri MA Moyano RD Characterization of a *Mycobacterium avium* subsp. *avium* operon associated with virulence and drug detoxification. Biomed Res Int (2014) 2014:809585. 10.1155/2014/809585 24967408 PMC4055363

[60] Tateishi Y Hirayama Y Ozeki Y Nishiuchi Y Yoshimura M Kang J Virulence of *Mycobacterium avium* complex strains isolated from immunocompetent patients. Microb Pathog (2009) 46:6–12. 10.1016/j.micpath.2008.10.007 19013228

[61] Li YJ Danelishvili L Wagner D Petrofsky M Bermudez LE. Identification of virulence determinants of *Mycobacterium avium* that impact on the ability to resist host killing mechanisms. J Med Microbiol (2010) 59:8–16. 10.1099/jmm.0.012864-0 19745033 PMC2887559

[62] Ostland VE Watral V Whipps CM Austin FW St-Hilaire S Westerman ME Biochemical, molecular, and virulence characteristics of select *Mycobacterium marinum* isolates in hybrid striped bass *Morone chrysops* x *M. saxatilis* and zebrafish Danio rerio. Dis Aquat Organ (2008) 79:107–18. 10.3354/dao01891 18500027

[63] Agdestein A Jones A Flatberg A Johansen TB Heffernan IA Djonne B Intracellular growth of *Mycobacterium avium* subspecies and global transcriptional responses in human macrophages after infection. BMC Genomics (2014) 15:58. 10.1186/1471-2164-15-58 24450835 PMC3906092

